# Treatment of Diseased Gums

**Published:** 1874-10

**Authors:** 


### TREATMENT OF DISEASED GUMS.

It was our privilege a short time ago, in an interview with our esteemed professional brother, Dr. Riggs, of Hartford, Ct., to learn from him his views of the various phases of diseased gums, that are so often presented to the dentist, and that constitute a very material and important part in his daily practice; and his method of treatment. His treatment in almost all such cases is chiefly of an operative character. The treatment is based upon the supposition, that almost if not all cases of disease of the gums, as ordinarily presented, are produced by local causes that consist mainly in deposits upon the necks of the teeth, primarily, causing separation of the gum gradually from, the roots and further deposit upon them; and accompanying this inflammation, tumefaction, suppuration and wasting of the tissue.

The doctor's treatment of these conditions consists simply in the removal of every particle of foreign substance from the roots of the teeth, not only that upon and in the vicinity of the necks of the teeth and beneath the margin of the gums, but extending upon the roots as far as any separation between them and the gum or alveolus is effected. He has devised and had made a set of instruments, six in number, very perfectly adapted to the purpose. They are so formed as easily to reach any point of any root in the mouth, when a separation of the surrounding tissue is effected.

The execution of the method consists in subjecting every such exposed surface of the roots to a thorough dressing with those instruments. The presumption is that the surface of every root, that becomes detached from its surrounding tissue, very soon affords lodgment for foreign substances, that would not only form an absolute barrier to a reunion, but be productive of disease of the gum and too often of the process as well; and the only method of removing the disease, and securing the attachment of the teeth is the removal of the foreign substances that may be upon the roots. It is not necessary that this should be dense or hard material, as the calcareous deposits, that are so often found, but soft glutinous, acid matter, will be more offensive and deleterious than deposits of dense calcareous matter.

The entire removal of every vestige of all foreign matter from the surfaces of the roots that should have an attachment is the plan and rationale of this treatment.

The instruments are so formed that round smooth surfaces are presented to the gums during the operation, so that very little interference is made with the gum. The abrading and breaking up the abnormal surface of the gum next to the root is oftentimes an important part of the work.

